# Retained NK Cell Phenotype and Functionality in Non-alcoholic Fatty Liver Disease

**DOI:** 10.3389/fimmu.2019.01255

**Published:** 2019-06-04

**Authors:** Natalie Stiglund, Kristina Strand, Martin Cornillet, Per Stål, Anders Thorell, Christine L. Zimmer, Erik Näslund, Silja Karlgren, Henrik Nilsson, Gunnar Mellgren, Johan Fernø, Hannes Hagström, Niklas K. Björkström

**Affiliations:** ^1^Department of Medicine Huddinge, Center for Infectious Medicine, Karolinska Institutet, Karolinska University Hospital, Stockholm, Sweden; ^2^Hormone Laboratory, Haukeland University Hospital, Bergen, Norway; ^3^Mohn Nutrition Research Laboratory, Department of Clinical Science, University of Bergen, Bergen, Norway; ^4^Department of Upper GI, Karolinska University Hospital, Stockholm, Sweden; ^5^Department of Medicine Huddinge, Karolinska Institutet, Stockholm, Sweden; ^6^Department of Surgery, Ersta Hospital, Stockholm, Sweden; ^7^Department of Clinical Sciences, Danderyd Hospital, Karolinska Institutet, Solna, Sweden

**Keywords:** natural killer cells, liver immunology, adipose tissue immunology, NAFLD, obesity

## Abstract

Non-alcoholic fatty liver disease (NAFLD), and the progressive stage non-alcoholic steatohepatitis (NASH), is the predominant cause of chronic liver disease globally. As part of the complex pathogenesis, natural killer (NK) cells have been implicated in the development of liver inflammation in experimental murine models of NASH. However, there is a lack of knowledge on how NK cells are affected in humans with this disease. Here, we explored the presence of disease-specific changes within circulating and tissue-resident NK cell populations, as well as within other major immune cell subsets, in patients with liver biopsy-confirmed NAFLD. Using 18-color-flow cytometry, substantial changes were observed in certain myeloid populations in patients as compared to controls. NK cell numbers, on the other hand, were not altered. Furthermore, only minor differences in expression of activating and inhibitory NK cell receptors were noted, with the exception of an increased expression of NKG2D on NK cells from patients with NASH. NK cell differentiation remained constant, and NK cells from these patients retain their ability to respond adequately upon stimulation. Instead, considerable alterations were observed between liver, adipose tissue, and peripheral blood NK cells, independently of disease status. Taken together, these results increase our understanding of the importance of the local microenvironment in shaping the NK cell compartment and stress the need for further studies exploring how NASH affects intrahepatic NK cells in humans.

## Introduction

NK cells are an important part of innate immunity where they participate in the defense against viral infections and in tumor surveillance (1) Upon activation, NK cells perform cytotoxicity by the release of cytolytic granules. They can also contribute to a pro-inflammatory environment through the production of cytokines and chemokines such as interferon-γ (IFN-γ) and tumor necrosis factor (TNF) (1). As part of the innate immune system, NK cells were believed to retain a static phenotype during their life span with little evidence of differentiation except for transition from CD56^bright^ to CD56^dim^ NK cells (2). However, this view has been revised in the last decade and it is now clear that NK cells gradually undergo directed differentiation even after they have reached the CD56^dim^ stage (2). In addition to their presence in the circulation, NK cells are found in numerous peripheral tissues and are especially enriched in the liver and uterus where they comprise up to 30 and 45%, respectively, of all lymphocytes (3, 4). However, compared to circulating NK cells, less is known regarding NK cells residing in tissues. In relation to the liver, studies have in recent years shown the importance of NK cells in the pathogenesis and clearance of chronic viral hepatitis infections in humans (5). However, the role of NK cells in many other liver diseases remains elusive.

Non-alcoholic fatty liver disease (NAFLD) is the most common chronic liver disease worldwide. A sub-group of NAFLD patients develop chronic inflammation in the liver, which over time can lead to liver fibrosis. This stage of the disease is known as non-alcoholic steatohepatitis (NASH) (6) and these patients are at risk of developing liver cirrhosis, liver failure, and hepatocellular carcinoma (HCC) (7). The increased rates of obesity in many countries has contributed to the drastic global increase in NAFLD prevalence in recent years (8). Thus, NAFLD complications, such as NASH, liver cirrhosis, and HCC, are posing a significant challenge to health care systems worldwide (6). Several theories exist as to why liver inflammation develops in patients with NAFLD. In more detail, disease development is believed to be influenced by an interplay between genetic and environmental factors, ranging from disturbances in lipid storage and metabolism, changes in dietary patterns and microbiota, to perturbed immune activation (6). However, the exact mechanisms as to why some patients progress in their disease whilst others do not still remain elusive.

Interestingly, murine models have revealed the importance of innate immunity, and in particular NK cells, during NASH-development (9). Many NK cell ligands are up-regulated in the liver of mice with NASH and this is followed by influx of activated cytotoxic NK cells (10, 11). Furthermore, NK cells can, via Tumor necrosis factor (TNF)–related apoptosis-inducing ligand (TRAIL) production, promote a pro-inflammatory state in the steatotic liver and by this mechanism contribute to progression toward steatohepatitis (10). In addition, NK cell activation in response to IL-15 promotes NASH-development in mice (12) and NK cells are also thought to play an important role in regulating fibrosis development in NASH (13, 14). In addition, obesity itself can also alter NK cell phenotype, metabolism, and function (15–18). Indeed, several recent studies in mice have suggested NK cells to be important for development of insulin resistance (19–21). However, there is a lack of knowledge on how NK cells are affected in humans with NASH, both with respect to circulating NK cells as well as to NK cells residing in metabolically active tissues such as liver and adipose tissue. To address this, we here performed an in-depth phenotypic and functional analysis of circulating NK cells in NAFLD patients as well as explored the NK cell compartment in liver and adipose tissue of these individuals.

## Materials and Methods

### Clinical Cohorts

Several clinical cohorts were included in the current study. All studies were approved by the regional ethics committee in Stockholm (Dnr's: 2010/678-31/3, 2006/971-31/1, 2006/229-31/3, and 2014/979-31/1) and oral and written informed consent was obtained from all participants. First, peripheral blood samples were obtained from 26 patients with liver biopsy-confirmed NAFLD from the out-patient clinic at the Upper GI Tract Department, Karolinska University Hospital, Stockholm, Sweden. See Table 1 for detailed patient characteristics. Secondly, as controls, peripheral blood from 15 healthy blood donors was collected from the blood bank at the Karolinska University Hospital, Stockholm, Sweden. Inclusion criteria in controls were normal body-mass index (BMI), normal liver enzymes [alanine aminotransferase (ALT) and aspartate aminotransferase (AST)], and no history of type 2 diabetes. Thirdly, in order to assess tissue resident NK cells, peripheral blood as well as liver and adipose tissue biopsies were collected from 26 patients undergoing laparoscopic gastric bypass surgery for morbid obesity at Danderyd and Ersta Hospitals in Stockholm. All patients had a BMI above 35 and had no previous history of liver disease. Included patients were not prescribed any low-calorie pre-surgery diet since this would influence degree of liver steatosis and possibly liver inflammation. A fraction of obtained liver biopsies was used for clinical scoring of liver histology according to the NAFLD activity score (NAS) and fibrosis stage. Based on the severity of the liver histology, patients were divided into three groups; patients with normal liver histology, patients with liver steatosis only (non-alcoholic fatty liver, NAFL), and patients with liver inflammation (NASH). See Table 2 for more detailed characteristics on this patient cohort.

**Table 1:** NAFLD cohort characteristics.

	**Healthy controls**	**NAFL^a^**	**NASH^b^**
Subjects	15	11	15
Age, mean (years)	54	56	58
Male, n	10 (67%)	8 (73%)	9 (60%)
Female, n	5 (33%)	3 (27%)	6 (40%)
BMI^c^ mean (kg/m^2^)	24	35	31
ALT^d^, mean (μkat/L)	NA	0.51	0.75
NAFLD Activity Score, median (range)	NA	3 (1–4)	5 (5–7)
Steatosis (0–3), median (range)	NA	1 (1–2)	2 (1–3)
Lobular inflammation (0–3), median (range)	NA	1 (1–3)	2 (1–3)
Hepatocyte ballooning (0–2), median (range)	NA	0 (0–1)	2 (1–2)
Fibrosis score (0–4)^e^, median	NA	0 (0–1)	2 (0–3)

a Non-alcoholic fatty liver.

b Non-alcoholic steatohepatitis.

c Body Mass Index.

d Alanine aminotransferase.

e Fibrosis score according to Kleiner et al. (22).

*NA, not applicable*.

**Table 2:** Bariatric surgery cohort characteristics.

	**Non-NAFLD**	**NAFL^a^**	**NASH^b^**
Subjects	8	10	5
Age (year), mean	43	45	43
Male	1 (12.5%)	3 (30%)	0 (0%)
Female	7 (87.5%)	7 (70%)	5 (100%)
BMI^c^, mean (kg/m^2^)	37	37	37
ALT^d^ (μkat/L), mean	0.37	0.51	0.75
P-Insulin (IU^e^), mean	14.3	19.8	36.0
P-Glucose (mmol/L), mean	5.6	6.4	6.5
HOMA-IR^f^, mean	3.6	5.7	10.5
NAFLD Activity Score, median (range)	0 (0)	3.5 (1–4)	5 (5)
Steatosis (0–3), median (range)	0 (0)	1 (1–3)	2 (1–2)
Lobular inflammation (0–3), median (range)	NA	1 (0–2)	2 (1–2)
Hepatocyte ballooning (0–2), median (range)	NA	1 (0–2)	1 (1–2)
Fibrosis score (0-4)^g^, median (range)	1 (0–2)	1 (0–2)	2 (1–3)

a Non-alcoholic fatty liver.

b Non-alcoholic steatohepatitis.

c Body Mass Index.

d Alanine aminotransferase.

e International Units.

f Homeostatic model assessment—insulin resistance.

g *Fibrosis score according to Kleiner et al. (22)*.

### Isolation of PBMC From Blood Samples

Peripheral blood mononuclear cells (PBMC) were isolated from blood samples using density gradient centrifugation. Briefly, whole blood was diluted with phosphate buffered saline (PBS; Invitrogen, USA), carefully layered on top of Ficoll-Hypaque (GE Healthcare, UK), and centrifuged. The leukocyte layer was extracted, carefully washed, and cryopreserved in freezing medium [90% heat-inactivated fetal bovine serum (FBS; Sigma-Alderich, USA) and 10% dimethyl sulfoxide (DMSO; Life Technologies)] until flow cytometry experiments were performed.

### Isolation of Immune Cells From Liver and Adipose Tissue

The core liver biopsies were collected during surgery directly into complete cell medium [Hyclone RPMI (Invitrogen), 10% FBS, and 1 mM L-glutamine (Invitrogen)] and kept on ice until same-day processing. Tissue pieces were mechanically dissociated, followed by enzymatic digestion in collagenase II for 30 min at 37°C, and filtered through a 100 μm filter, before flow cytometry staining.

### Antibody Staining Protocol

Cryopreserved PBMC were thawed in a 37°C water bath, immediately transferred to cell medium, washed, and resuspended in complete cell medium. Three million PBMC were stained in each test. Flow cytometry primary and secondary stainings were performed in flow buffer (PBS with 2 mM EDTA and 2% FBS) for 20 min in the dark at room temperature. After staining, cells were fixed for 15 min in the dark at room temperature using the Fix/Perm solution (eBioscience, USA). Finally, Fix/Perm solution was washed away, cells were resuspended in flow buffer, and kept at 4°C in the dark until they were acquired on the flow cytometer. For intracellular stainings, cells were permeabilized in Fix/Perm solution for 45 min and then stained for 30 min in permeabilization buffer (eBioscience, USA), diluted 1:10 with MQ water, before being washed and analyzed. All samples were run on an 18-color LSRFortessa (BD Biosciences, USA) equipped with 355, 405, 488, 561, and 639 nm lasers.

### Functional Experiments

NK cell degranulation and cytokine production were assessed by co-culture experiments with target cells. PBMCs were pre-stimulated overnight with IL-12 (10 ng/ml, Peprotech) and IL-18 (100 ng/mL, Medical & Biological Laboratories) and then K562 cells were added at a 1:10 ratio. One hour after addition of target cells, Golgi plug (Brefeldin A, BD Biosciences) and Golgi stop (Monesin, BD Biosciences) were added and the assay was continued for an additional 5 h. NK cells were then stained for analysis using flow cytometry.

### Microscopy

Liver biopsy specimens obtained from NAFL/NASH patients and patients undergoing gastric bypass surgery were stained with hematoxylin and eosin and graded in a blinded fashion by an experienced hepatologist according to the NAFLD activity score (NAS) (23). In addition, liver biopsies were stained with Sirius red to evaluate liver fibrosis and scored according to Kleiner on a 0–4 scale (23).

### Flow Cytometry Analysis

To avoid bias from intra-experimental variability that could affect the flow cytometry analysis, samples from both healthy donors and patients were analyzed in each experiment. Acquired data was compensated using a compensation matrix generated based on antibody-stained control beads and analyzed using FlowJo Version 9.6.4 (Treestar, USA). Apart from conventional flow cytometry analysis, Barnes-Hut stochastic neighbor embedding (SNE) analysis, using an in-house built script (24) in R (The R Foundation for Statistical Computing), was performed in order to visualize potential differences not present in two-dimensional space. For SNE-analysis, 1,000 CD56^dim^ NK cells or 500 CD56^bright^ NK cells from each donor was included. The data was clustered based on median fluorescence intensity (MFI) of the following markers: CD16, CD25, CD44, CD49a, CD56, CD57, CD69, CD107a, HLA-DR, IFN-γ, KIRs, MIP-1β, NKG2A, NKG2C, and TNF.

### Staining of Primary Hepatocyte

Primary human hepatocytes were isolated from three organ donors whose livers were not used for liver transplantation, acquired from the Karolinska University Hospital, using a protocol previously described (25). The cells were then stained fresh with antibodies against MICA, MICB, HLA-ABC, CD155, ULBP-1, ULBP-2, and ULBP-3 conjugated with PE and analyzed on a BD FACS Accuri.

### Quantification of Soluble MICA and MICB by ELISA

MICA and MICB solid-phase sandwich ELISAs (enzyme-linked immunosorbent assay, Thermofisher) were performed according to the manufacturer instructions. Briefly, human sera were incubated 2, 5, and 2 h respectively diluted two times in provided buffers (capture phase). After washing, biotin-conjugated anti-MICA and MICB antibodies were incubated for 1 h and streptavidin-HRP was added after washing. Unbound streptavidin-HRP was removed by washing and substrate solution reactive with HRP was added. The reaction was stopped by acid after 30 min and absorbance measured at 450 nm.

### Statistical Analysis

Data were analyzed using Prism Version 6.0 b (GraphPad Software Inc.; USA). The Mann–Whitney *U*-test or *t*-tests were used depending on if data sets were normally distributed, using D'Agostino-Pearson omnibus normality test. The threshold for statistical significance was set to α < 0.05. For analysis of NK cell population diversity, Simpson Diversity Index (SDI) was calculated as previously described (26).

## Results

### Global Assessment of Immune Cells in NAFL and NASH

**Figure 1:**
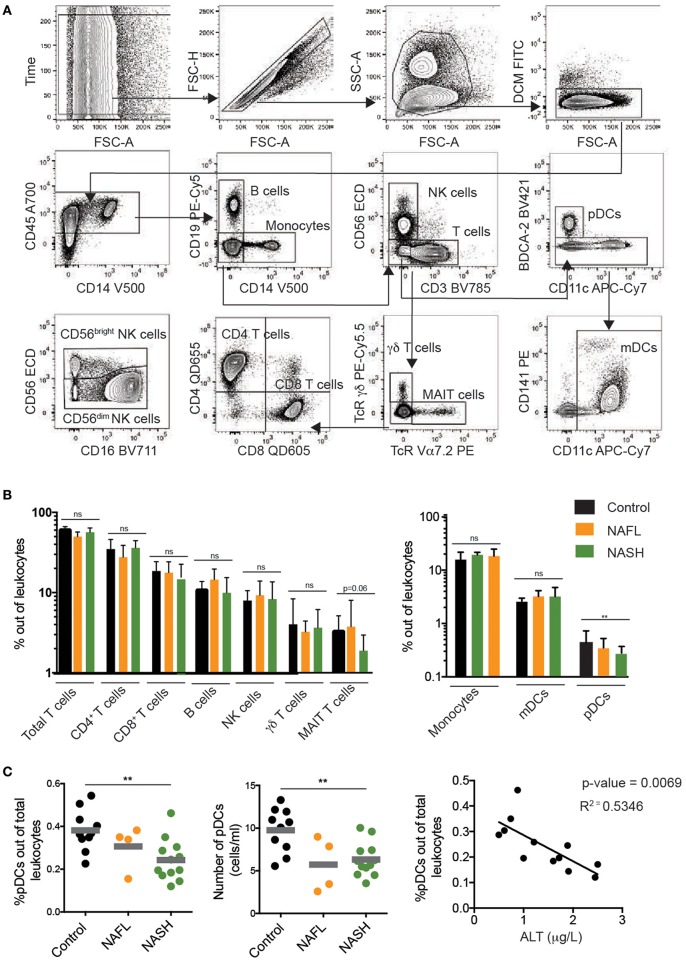
Immunophenotyping of major peripheral blood immune cell subsets in NAFL and NASH. **(A)** Flow cytometry gating scheme used to identify the investigated immune cell subsets. Arrows indicate the sequence of gating. **(B)** Summary data for the frequency out of total leukocytes for the indicated immune cell populations in healthy controls (*n* = 10), NAFL (*n* = 4), and NASH (*n* = 11) patients. **(C)** Summary data of pDC frequency out of total leukocytes (left), absolute counts of pDCs (middle), and correlation between pDC frequency and ALT (right) in the indicated patient groups. Bars in **(B,C)** represent mean and error bars show SEM. ^**^p < 0.01.

To determine the impact of NAFL and NASH on the peripheral blood immune cell compartment, a broad profiling of major lymphoid and myeloid immune cells in patients and controls was initially performed (Figure 1A). Whereas, few differences were noted among CD4, CD8, and γδ T cells, a trend toward a decline in MAIT cells was observed in NASH patients as compared to NAFL patients and healthy controls (Figure 1B). A decrease of MAIT cells in NASH is in line with recent literature (27). Regarding NK cells, neither frequency (Figure 1B) nor absolute numbers (data not shown) were affected by the presence of obesity, NAFL, or NASH. With respect to the myeloid immune cell compartment few differences were observed for monocytes and myeloid DCs. However, a decline in the frequency of plasmacytoid DCs (pDCs) was noted in NASH patients (Figure 1C). Also, the absolute numbers of pDCs were decreased and the loss of pDCs correlated inversely with the degree of liver damage measured as serum alanine transferase levels (Figure 1C). Taken together, although alterations could be observed in certain innate immune cell subsets, the overall size of the peripheral blood NK cell compartment remained unaltered in NAFL and NASH.

### Upregulation of NKG2D on NK Cells From NASH Patients

**Figure 2:**
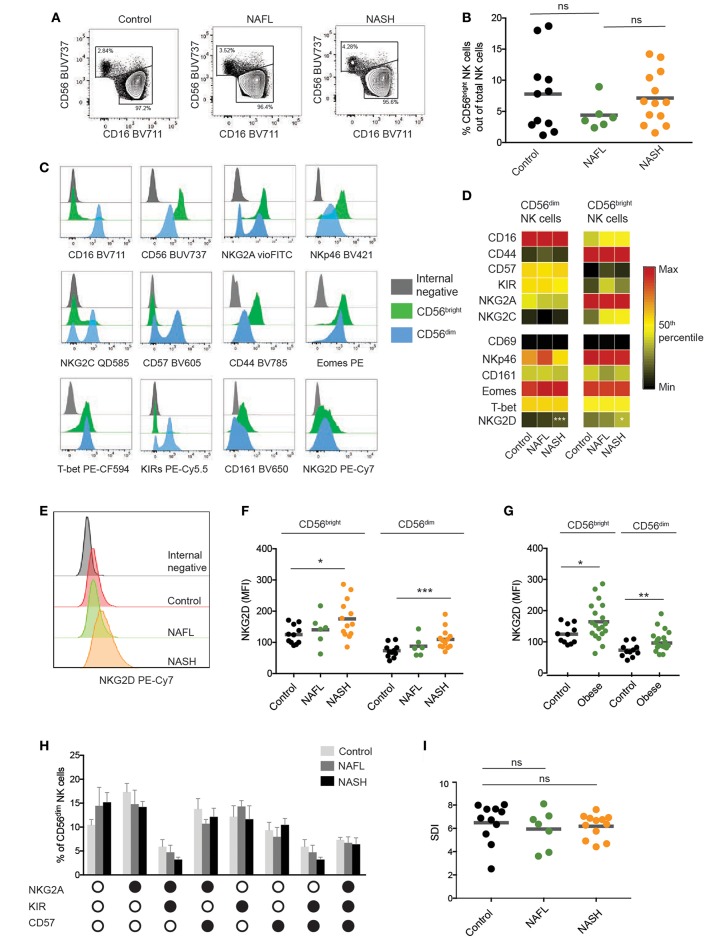
Phenotypic characterization of circulating NK cells from NAFLD patients. **(A)** Representative flow cytometry plots of NK cells from healthy, NAFL, and NASH patients. **(B)** Frequency of CD56^bright^ NK cells out of total NK cells in peripheral blood of healthy controls (*n* = 13), NAFL patients (*n* = 9), and NASH patients (*n* = 16). **(C)** Representative histograms for the indicated markers on CD56^bright^ and CD56^dim^ NK cells as well as internal negative control. The plots represent stainings from one healthy donor. **(D)** Heat map depicting the mean frequency of NK cells expressing CD16, CD44, CD57, KIRs, NKG2A, and NKG2C as well as the mean MFI of CD69, NKp46, CD161, Eomes, T-bet, and NKG2D on CD56^dim^ and CD56^bright^ NK cells for the indicated groups. **(E)** Representative histogram of NKG2D expression on NK cells from healthy control, NAFL, and NASH patients respectively. **(F,G)** Scatter plots of NKG2D MFI on CD56^dim^ and CD56^bright^ NK cells from the indicated groups. In **(F)**, healthy controls (*n* = 11), NAFL (*n* = 6), and NASH (*n* = 13) patients, in **(G)** healthy controls (*n* = 11), obese individuals (*n* = 18). **(H)** Bar graph showing the frequency of CD56^dim^ NK cells that express different combination of NKG2A, KIRs, and CD57. Black circles indicate presence of the marker and white circles no expression. **(I)** Inverse Simpson Diversity index (SDI) analysis for healthy (*n* = 11), NAFL (*n* = 7), and NASH patients (*n* = 12). The Mann–Whitney *U*-test was used for comparison between groups. Bars in **(B,F,G,I)** show mean, error bars in H represent SEM. ^*^p < 0.05, ^**^p < 0.01, ^***^p < 0.001.

Since NK cells are far from a homogeneous population, a more in-depth immune-phenotyping of activating and inhibitory receptors on circulating NK cells was performed. The CD56^dim^ to CD56^bright^ NK cell relationship was unaffected in NAFL and NASH (Figures 2A,B). Next, we simultaneously assessed expression of 12 surface and intracellular markers on the NK cells (Figure 2C). As expected, CD56^dim^ NK cells expressed higher levels of NKG2C, KIRs, and CD57, while CD56^bright^ NK cells had a higher expression of NKG2A, CD161, CD44, and NKp46 (Figures 2C,D). Surprisingly, neither the degree of NAFLD disease severity (Figure 2D) nor presence of obesity (data not shown) had a detectable effect on the NK cell receptor repertoire on circulating NK cells, with the exception for expression of the activating receptor NKG2D. In more detail, both CD56^bright^ and CD56^dim^ NK cells from patients with NASH expressed significantly higher levels of NKG2D on their surface (Figures 2E,F). This was also observed when comparing normal weight with obese individuals (Figure 2G). However, since NK cells from NAFL patients had close to normal levels of NKG2D (Figure 2F), this would suggest that increased expression of NKG2D primarily associated with NASH. Furthermore, this increase was specific to NK cells since it was not observed on T cells from the same patients (data not shown). To dissect the role of NKG2D more in-depth in relation to the liver and NAFL we assessed presence of NKG2D-ligands. No difference in levels of soluble MICA and MICB was noted in patients as compared to controls (data not shown). Furthermore, primary human hepatocytes from healthy organ donors were negative for NKG2D-ligands whereas CD155 and HLA class I was expressed (Supplementary Figure 1).

Finally, we assessed NK cell differentiation, as determined by the expression of NKG2A, KIRs, and CD57, as well as NK cell diversity by calculating Simpson diversity index (SDI) (28, 29). However, both of these metrics for NK cell compartment composition remained unaltered in NAFL and NASH patients as compared to healthy controls (Figures 2H,I). In summary, the phenotype of the circulating NK cell population remains unaffected by NAFL and NASH with the exception of NKG2D being upregulated in patients with NASH.

### Functional Capacity of Peripheral NK Cell Subsets in NAFL and NASH

**Figure 3:**
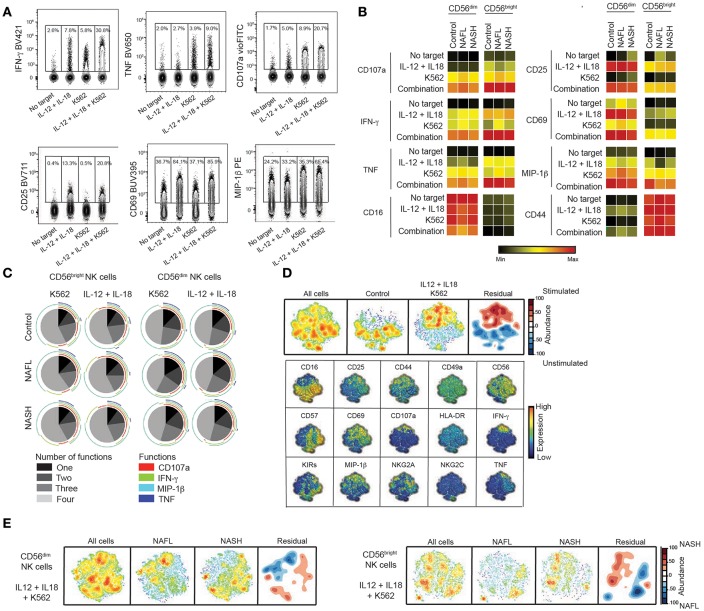
Retained functional capacity of circulating NK cells in NAFLD. **(A)** Representative concatenated flow cytometry plots showing CD56^dim^ NK cell responses following the indicated stimulations. **(B)** Heat map summarizing the mean frequency of responding NK cells for the measured functions and indicated stimulations in healthy controls (*n* = 15), NAFL patients (*n* = 10), and NASH patients (*n* = 16). **(C)** Multifunctional analysis of the combination of degranulation (CD107a) and cytokine production (IFN-γ, MIP-1β, and TNF) responses for IL-12 + IL-18 stimulation and/or co-culture with K562 target cells. **(D)** SNE plots of total cells from healthy donors subsequently divided in stimulated and un-stimulated cells. The residual plot highlights the decreased (blue) or more highly expressed (red) areas in the stimulated cells. Underneath, individual plots of the expression of 15 single markers are shown. **(E)** SNE plots depicting the difference between CD56^dim^ (left) and CD56^bright^ (right) NK cells from NAFL and NASH patients after IL-12 + IL-18 + K562 stimulation where the density plots highlight the specific changes in NASH patients.

Having determined the NK cell phenotype in peripheral blood, we next evaluated the functional capacity of NK cells in NAFL and NASH. To this end, NK cells were stimulated with cytokines (IL-12+IL-18) and/or K562 target cells and production of IFN-γ, TNF, MIP-1β, upregulation of CD107a, CD69, CD44, and CD25, as well as downregulating of CD16 as a consequence of activation was measured using flow cytometry (Figure 3A). As expected, stimulation with cytokines led to high levels of IFN-γ being produced as well as strong upregulation of CD25 and CD69 whereas K562 cell stimulation yielded a robust degranulation response and elevated levels of TNF and MIP-1β (Figures 3A,B). When assessing single functional responses, NK cells from patients with NASH had a similar capacity to degranulate, produce cytokines, and upregulate activation markers as their NAFL counterparts and the healthy controls (Figure 3B). Also, the ability of NK cells to perform multiple functions was retained in both NAFL and NASH patients as compared to healthy controls (Figure 3C).

The NK cell compartment consists of many different subpopulations and NK cells can produce a multitude of functions in a variegated fashion. Although single or multifunctional analysis of NK cell functional responses revealed no considerable alterations in function when comparing NASH with healthy controls it is plausible that functional difference might exist in multivariate space not allowing identification by conventional flow cytometry gating. To address this, we performed a SNE analysis of the NK cell functional responses and first generated SNE maps of responding compared to non-responding NK cells (Figure 3D). These SNE maps segregated considerably (Figure 3D, residual map) and by projecting the differences onto the single parameters that formed the basis of the SNE map the pattern of NK cell responses in multidimensional space could be revealed (Figure 3D, lower panels). In more detail this shows how certain NK cell subpopulations responded with many functions and others only with single function. Together, this validated the analysis approach and allowed us to compare patients with healthy controls (Figure 3E and data not shown). Although the residual plots revealed difference when comparing responding NK cells from healthy controls with NAFL or NASH patients (data not shown) or NAFL patients with NASH patients (Figure 3E), these differences could not be attributed to a specific phenotype when the residual plot was projected onto the single parameters (data not shown). This suggests that also in multi-dimensional space, the NK cell response was unaltered in NAFL and NASH.

### Characterization of NK Cells in Adipose and Liver Tissue

**Figure 4:**
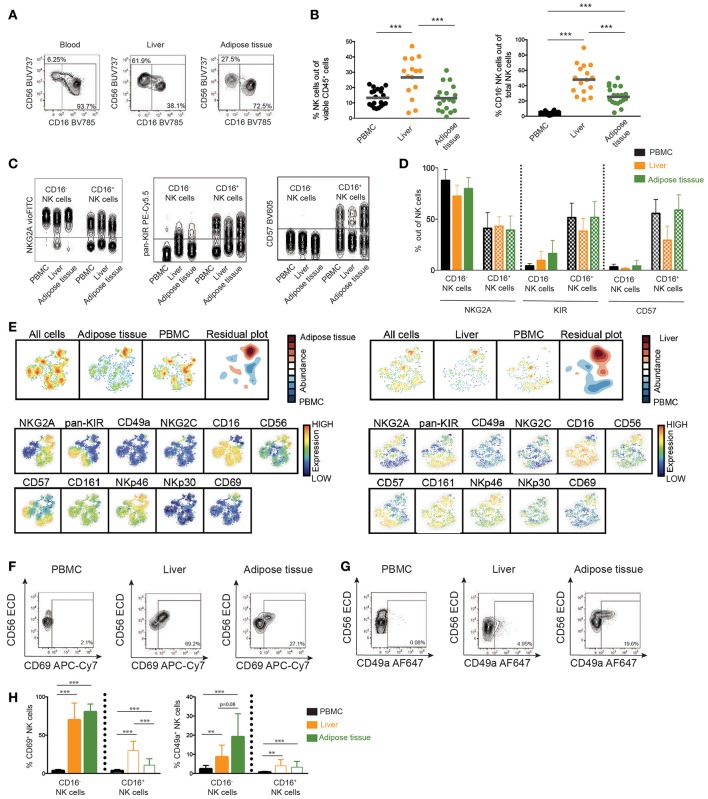
Phenotype of NK cells in liver and adipose tissue. **(A)** Representative flow cytometry plots of NK cells derived from blood, liver, and adipose tissue. **(B)** Scatter plot of the frequency of total NK cells out of viable leukocytes and CD16^−^ NK cells out of total NK cells in peripheral blood, liver, and adipose tissue. PBMC (*n* = 22), adipose tissue (*n* = 19), and liver (*n* = 15). **(C)** Representative concatenated flow cytometry plots showing expression of differentiation markers NKG2A, KIRs, and CD57 on CD16^−^ or CD16^+^ NK cells in blood, liver, and adipose tissue. **(D)** Bar graphs showing frequency of cells expressing the indicated differentiation markers in CD16^−^ or CD16^+^ NK cells from blood, liver, and adipose tissue. PBMC (*n* = 22), adipose tissue (*n* = 19), and liver (*n* = 15). **(E)** SNE plot of total NK cells from adipose tissue (left) or liver (right) compared to peripheral blood. The residual plot highlights the decreased (blue) or more highly expressed (red) areas within tissue NK cells. **(F,G)** Representative flow cytometry plots showing CD69 and CD49a expression on total NK cells from the indicated tissues. **(H)** Expression of CD69 and CD49a on CD16^−^ and CD16^+^ NK cells in peripheral blood, liver, and adipose tissue. In H, CD69 on PBMC (*n* = 22), adipose tissue (*n* = 18), and liver (*n* = 15), CD49a on PBMC (*n* = 9), adipose tissue (*n* = 9), and liver (*n* = 7). Bars in **(B,D,H)** represent mean and error bars in **(D,H)** show SEM. ^**^p < 0.01, ^***^p < 0.001.

To this end, characteristics of circulating NK cells have been analyzed. However, NAFL and NASH are diseases of the liver and also tightly coupled to obesity, adipose tissue dysregulation, and reduced insulin sensitivity. Thus, the analysis was focused on NK cells from liver and adipose tissue. In line with previous literature (9), NK cells were enriched in the liver compared to peripheral blood (Figures 4A,B) and a sizeable population of NK cells could also be detected in visceral adipose tissue. Furthermore, both types of investigated tissue had a skewing in the distribution of CD56^bright^ and CD56^dim^ NK cells as compared to circulation with CD56^bright^ NK cells representing up to half of all NK cells in liver and adipose tissue (Figure 4B). Also, whereas the differentiation status of adipose tissue NK cells, with respect to expression of NKG2A, KIRs, and CD57, mirrored that of circulating NK cells, differences were observed especially within liver CD56^dim^ NK cells in expression of these markers (Figures 4C,D).

SNE-analysis of the NK cell compartment identified that NK cells derived from liver and adipose tissue displayed a unique phenotype compared to peripheral blood NK cells (Figure 4E). The major distinction between NK cells derived from circulation and NK cells derived from tissues was a higher expression of the tissue residency marker CD69 (Figure 4E). As a consequence of liver and adipose tissue containing larger populations of CD56^bright^ NK cells, the SNE analysis also identified higher expression of NKp46, NKG2A, and CD56 but lower expression of CD16, CD57, and KIRs within the population enriched in the tissues compared to in circulation (Figure 4D). This is in line with the overall phenotypic differences observed when comparing CD56^bright^ with CD56^dim^ NK cells (Figures 2C,D). Finally, we confirmed expression of tissue residency markers on liver and adipose tissue by conventional flow cytometry gating (Figures 4F,G). As expected, the expression was primarily confined to the CD56^bright^ NK cells. Interestingly, while CD69 was highly expressed in both liver and adipose tissue, CD49a was only found on a small fraction of CD56^bright^ NK cells in the liver whereas higher expression was noted in adipose tissue (Figures 4F–H). These results highlight the importance of the organ-specific microenvironment in shaping the local NK cell population and emphasize the importance of studying the tissue-resident compartment when trying to understand the pathogenesis of diseases affecting peripheral organs.

### The Phenotype of Tissue NK Cells Remains Unaltered in NAFL and NASH

**Figure 5:**
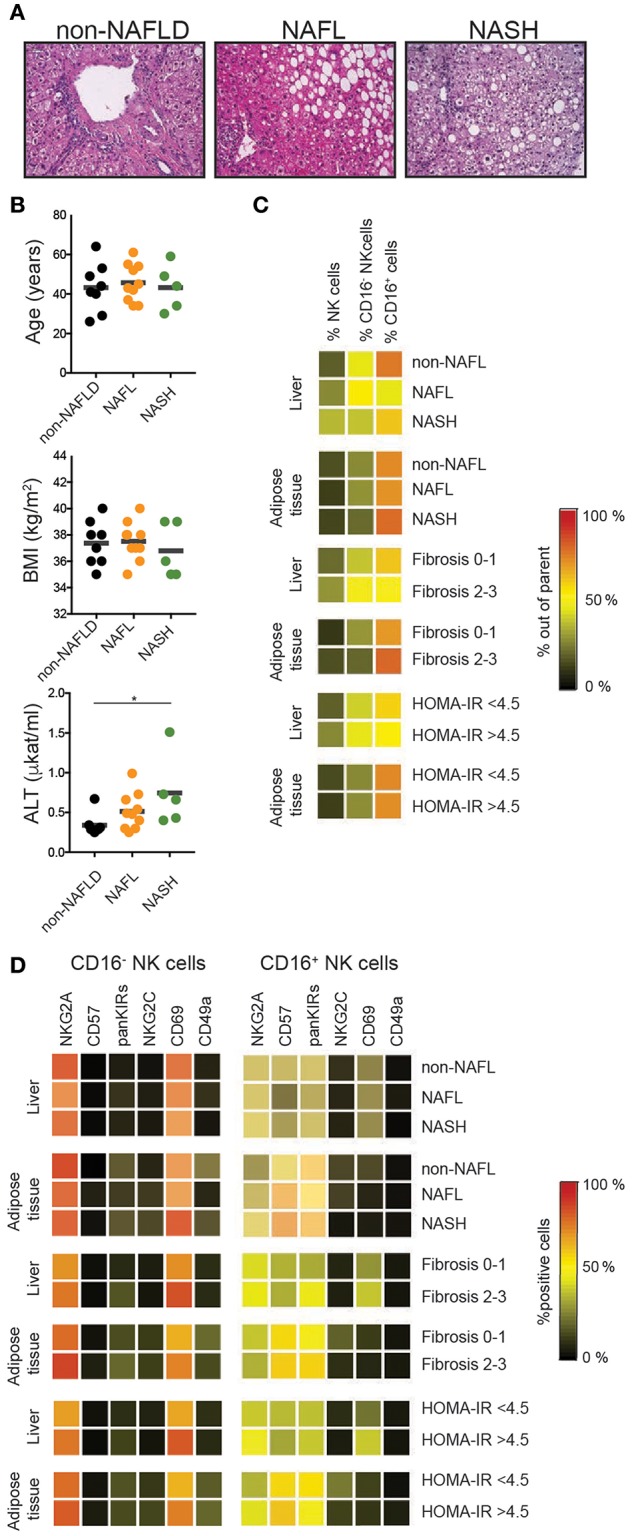
Tissue NK cells in relation to patient disease characteristics. **(A)** Representative hematoxylin and eosin stainings on normal, NAFL, and NASH liver sections. **(B)** Distribution of the patients in relation to age, BMI, and levels of alanine transferase (ALT) as a proxy for liver damage. **(C)** Heat map showing mean frequency of total, CD16^+^, and CD16^−^ NK cells for the indicated groups. **(D)** Heat map showing mean frequencies of NKG2A, CD57, KIRs, NKG2C, CD69, and CD49a expression on CD16^−^ and CD16^+^ NK cells for the indicated groups. ^*^p < 0.05.

Finally, we assessed the impact of NAFL and NASH disease stage and severity on the frequency and phenotype of liver and adipose tissue NK cells (Figure 5). Patients where data on tissue NK cells were available were stratified based on NAS score (non-NAFL which represented no steatosis present, NAFL, NASH), liver fibrosis (fibrosis score 0–1 vs. 2–3), and insulin sensitivity status (HOMA-IR < 4.5 vs. >4.5). Although NK cells were more abundant in liver (Figure 4B) their frequency remained unaffected by clinical stage (Figure 5C). Also, the frequency of NK cells in adipose was unaltered by NAFLD disease activity, level of fibrosis, and insulin sensitivity (Figure 5C). Finally, the NK cell phenotype was assessed. Since CD16^−^ and CD16^+^ NK cells display distinct phenotypes (Figures 2C,D, 4E–G), these were analyzed separately. For none of the investigated phenotypic markers, a clear link to the clinical parameters could be observed (Figure 5D).

Thus, whereas murine models suggest NK cells to play a distinct role in NAFL and NASH, both the human circulating and peripheral tissue NK cell compartments remain largely intact in these diseases.

## Discussion

NAFLD is the most common chronic liver disease worldwide and a subgroup of NAFLD patients develop chronic liver inflammation (NASH) with ensuing fibrosis and increased risk for HCC. Since little is known regarding NK cells in this disease and because NK cells are highly enriched in human liver, we here performed an extensive mapping of the NK cell compartment in NAFL/NASH using high-dimensional flow cytometry technology. We assessed the imprint of liver inflammation in NASH on circulating NK cells and show specific upregulation of the activating receptor NKG2D. In addition, by employing bariatric surgery as a human model, we also comprehensively mapped both liver and adipose tissue NK cells revealing substantial differences in NK cell population composition in-between tissues. However, no significant differences in the tissue-resident NK cell populations in patients with and without NAFL/NASH were detected.

NK cells have previously been investigated in other chronic liver diseases, especially in chronic viral hepatitis. These studies revealed that NK cells have decreased function in chronic hepatitis C (5, 30, 31). In obese individuals, decreased NK cell functionality combined with increased activation was reported (15, 16). A recent study showed that this reduced functionality was a result of metabolic paralysis of NK cells (18). However, our study detected no functional defects or changes in activation, despite both the in-depth and broad evaluation performed. These discrepancies might in part be based on methodological dissimilarities between the studies, with different target cells used but also on differing patient inclusion criteria. Indeed, our patients had, in general, mild fibrosis, with no patients suffering from cirrhosis, suggesting a more active inflammatory disease. This compared to other studies where many patients had cirrhosis (14) or higher BMI (15). Michelet et al identified functional defects as well as the loss of CD56^bright^ NK cells in obese patients (18). This is in line with a previous report (15), and common of these two studies was that included patients had BMI's of up to 50. Our cohort had a considerably lower average BMI of around 35. Together, this suggests that the functionality of NK cells and NK cell subset composition is retained in less severe obesity whereas alterations become evident in patients with morbid obesity. Atherosclerosis, hypertension, and adipose tissue inflammation are common comorbidities in NAFLD patients and thus potential confounding factors that should be controlled for. Our strategy to address this challenging issue was in one part to stratify patients into NAFL or NASH by the use of liver biopsies, considered the gold standard. In addition, we made a subgroup analysis based on the level of fibrosis and on insulin sensitivity. However, in none of these comparisons, neither in peripheral blood nor in liver or adipose tissue, any profound disease-related alterations in the NK cell populations could be observed. Of note, we focused our analysis on the major two subpopulations of NK cells: CD56^bright^ and CD56^dim^ NK cells. Apart from them, there are additional un-conventional NK cell subset, such as CD56^−^CD16^+^ and CD56^dim^CD16^−^ NK cells (32), that should be investigated in future studies.

A recent report demonstrated how chronic hepatitis C irreversibly causes decreased receptor repertoire diversity in circulating NK cells (26) Related changes have also been observed in chronic hepatitis D (33). Based on this, we wanted to investigate whether the chronic “non-infectious” inflammation in NAFLD could cause a similarly reduced diversity. However, NK cell repertoire diversity, determined by SDI, remained intact in NAFLD. Both NAFLD and chronic viral hepatitis are slowly developing liver diseases that take years to produce symptoms. However, many more changes in serum cytokines can be detected in hepatitis C patients as compared to NASH patients (34) and it is plausible that the general degree of inflammation in NASH is more low-grade as compared to chronic viral hepatitis. Thus, it might be that a larger inflammatory insult is needed in order to affect NK cell repertoire diversity.

In experimental models, NK cells have been shown to protect against liver fibrosis development, e.g., via NKp46-mediated macrophage activation (13) or by killing of hepatic stellate cells (HSCs) (35). Similar evidence exists in the human setting but primarily in relation to chronic viral hepatitis and fibrosis development (36). NKG2D is another NK cell surface receptor that may be protective against fibrosis development in mice by targeting activated HSCs (37). Furthermore, the NKG2D-ligands MICA and MICB are both upregulated in NASH-livers in mice (11). For NAFLD patients, level of fibrosis is an important predictor of long-term survival (38, 39). Interestingly, we could show that NK cells express higher levels of NKG2D in patients with NASH. This upregulated NKG2D expression could be a response to the increased hepatocyte stress, inflammation, and apoptosis that can be seen in NASH-livers (23). To determine if NKG2D influenced the degree of liver fibrosis in NAFLD patients, we compared levels of fibrosis to NKG2D expression on circulating NK cells but could not detect any association. This could be due to the fact that fibrosis is a dynamic process that occurs during many years in NAFLD. In the current study, we did not have the possibility to investigate NKG2D expression on intrahepatic NK cells. Future studies should assess this and also specifically evaluate the capacity of NK cells to target HSCs (or hepatocytes) via NKG2D in NAFLD.

Apart from having a protective role in fibrosis development, adipose tissue NK cells, or ILC1-like cells, have also been shown to augment insulin resistance in experimental murine models (19, 20, 40). In more detail, it has been proposed that NK cells sense stressed cells in adipose tissue, respond with IFN-γ production, in turn causing macrophage polarization toward a pro-inflammatory phenotype, which subsequently leads to insulin resistance (19, 20, 40). However, little is known about human adipose tissue NK cells in general and also how they relate to obesity, insulin resistance, and liver disease. In this regard, we assessed visceral adipose tissue NK cell frequency and phenotype. Adipose tissue contained a similarly large population of NK cells as found in peripheral blood. However, adipose tissue was clearly enriched for CD56^bright^CD16^−^ NK cells expressing tissue residency markers. This profile with enrichment of CD56^bright^ NK cells was similar to the phenotype of NK cells found in matched liver samples and also to NK cells in many other peripheral tissues in general (41). Interestingly, the specific subset of adipose tissue NK cells (or ILC1-like cells) that contribute to insulin resistance in mice express CD49a on their surface (21, 40). We here report that also human adipose tissue contains NK cells expressing CD49a. CD49a^+^ NK cells had a CD56^bright^CD16^−^ phenotype, which is different from liver CD49a^+^ NK cells (4), and were more prevalent in adipose tissue as compared to liver and peripheral blood. However, no link between the presence and levels of adipose tissue CD49a^+^ NK cells and the presence of insulin resistance was noted in the investigated patients. Within the scope of this study, only CD49a and CD69 was studied as tissue residency markers. There are a number of additional surface markers as well as transcription factors that are linked to tissue residency, which should be investigated in future studies.

Our study design, with liver and adipose tissue biopsies acquired during laparoscopic surgery, enabled us to uniquely study NK cells from different peripheral tissues within the same individual. This analysis revealed interesting features, emphasizing the different phenotypes of tissue-resident cells from distinct tissues. We could show that NK cell differentiation status differed not only between liver and peripheral blood, in line with previous reports (41), but also between liver and adipose tissue-derived NK cells. The pattern of tissue residency marker expression was also distinct between liver and adipose tissue NK cells within the same individual. These data emphasize the importance of the specific local microenvironment in influencing the shape of the NK cell population. This also shows the importance of, although cumbersome, studying tissue NK cells in different human disorders.

Limitations of our study have also to be considered. First, the target cell line used in the functional experiments consist of the leukemia cell line K562. While being the gold standard for assessment of NK cells function, due to its lack of MHC class I, it is not representative for the NAFLD setting. However, it does express NKG2D-ligands (42), which primary hepatocytes derived from organ donors did not (Supplementary Figure 1). Ideally, future studies should assess NK cell responsiveness against primary target cells derived from livers of NAFLD patients, such as hepatic stellate cells or hepatocytes. Second, in addition to the target cells derived from NASH-livers, future work should focus on the function of tissue-derived NK cells since this study, due to practical reasons, only assessed the function of circulating NK cells. Third, within the scope of this study, NKG2D expression was only studied on NK cells derived from peripheral blood. This might not be representative of NKG2D expression on NK cells derived from liver and adipose tissue. Fourth, this study does not address the NKG2D-ligand expression in NASH livers which, in combination with the NKG2D expression on hepatic NK cells, could provide interesting insights into the disease mechanisms of NASH. Thus, the exact role of NKG2D in NAFLD still remains to be elucidated.

In summary, we here performed a comprehensive assessment of peripheral blood and tissue NK cells in relation to NAFLD, the most common chronic liver disease worldwide. Surprisingly, despite a substantial literature from experimental model systems suggesting a role for NK cells in NASH, we found a largely intact NK cell compartment in the human setting. Instead, our study reveals significant differences in composition of the NK cell compartment between human peripheral tissues and, thus, illustrates the importance of understanding the local microenvironment in shaping the NK cell repertoire.

## Ethics Statement

This study was carried out in accordance with the recommendations of the Regional Ethics Committee of Stockholm, Stockholm, Sweden. All subjects gave oral and written informed consent in accordance with the Declaration of Helsinki. The protocol was approved by the Regional Ethical Review Board in Stockholm, Sweden.

## Author Contributions

NS designed the study, performed experiments, acquired and analyzed data, and drafted the manuscript. NB designed the study, performed data analysis, drafted the manuscript, and supervised the work. KS, MC, and CZ performed experiments and data analysis. JF and GM contributed to the data analysis, the discussion, and supervision. HH recruited patients and was instrumental in the histology assessments. PS and HH contributed with in-depth knowledge of NAFLD. AT, EN, SK, and HN recruited and sampled patients. All authors provided valuable contributions and insights into the manuscript.

### Conflict of Interest Statement

The authors declare that the research was conducted in the absence of any commercial or financial relationships that could be construed as a potential conflict of interest.

## Supplementary Material

Supplementary Figure 1 Expression of NKG2D ligands on primary human hepatocytes. Representative histogram of flow cytometry stainings of NKG2D-ligands as well as HLA-ABC and CD155, ligand of DNAM-1, on primary hepatocytes. One representative staining out of three.

## Abbreviations

ALT: alanine transferase
AST: aspartate transferase
BMI: body mass index
HCC: hepatocellular carcinoma
HOMA-IR: homeostatic model assessment
HSC: hepatic stellate cell
IFN-γ: interferon-gamma
IL: interleukin
ILC1: innate lymphoid cell group 1
KIR: killer cell immunoglobulin-like receptor
MAIT: mucosa-associated T
mDC: myeloid dendritic cell
NAFL: non-alcoholic fatty liver
NAFLD: non-alcoholic fatty liver disease
NASH: nonalcoholic steatohepatitis
NAS: NAFLD activity score
NK: natural killer
PBMC: peripheral blood mononuclear cell
pDC: plasmacytoid dendritic cell
SDI: Simpson diversity index
SNE: stochastic neighbor embedding
TRAIL: TNF-related apoptosis-inducing ligand
TNF: tumor-necrosis factor.
