# Supplementary figures and images for: Retained NK Cell Phenotype and Functionality in Non-alcoholic Fatty Liver Disease
Source: Front Immunol. 2019 Jun 4;10:1255. doi: 10.3389/fimmu.2019.01255 (PMC6558016; doi:10.3389/fimmu.2019.01255)

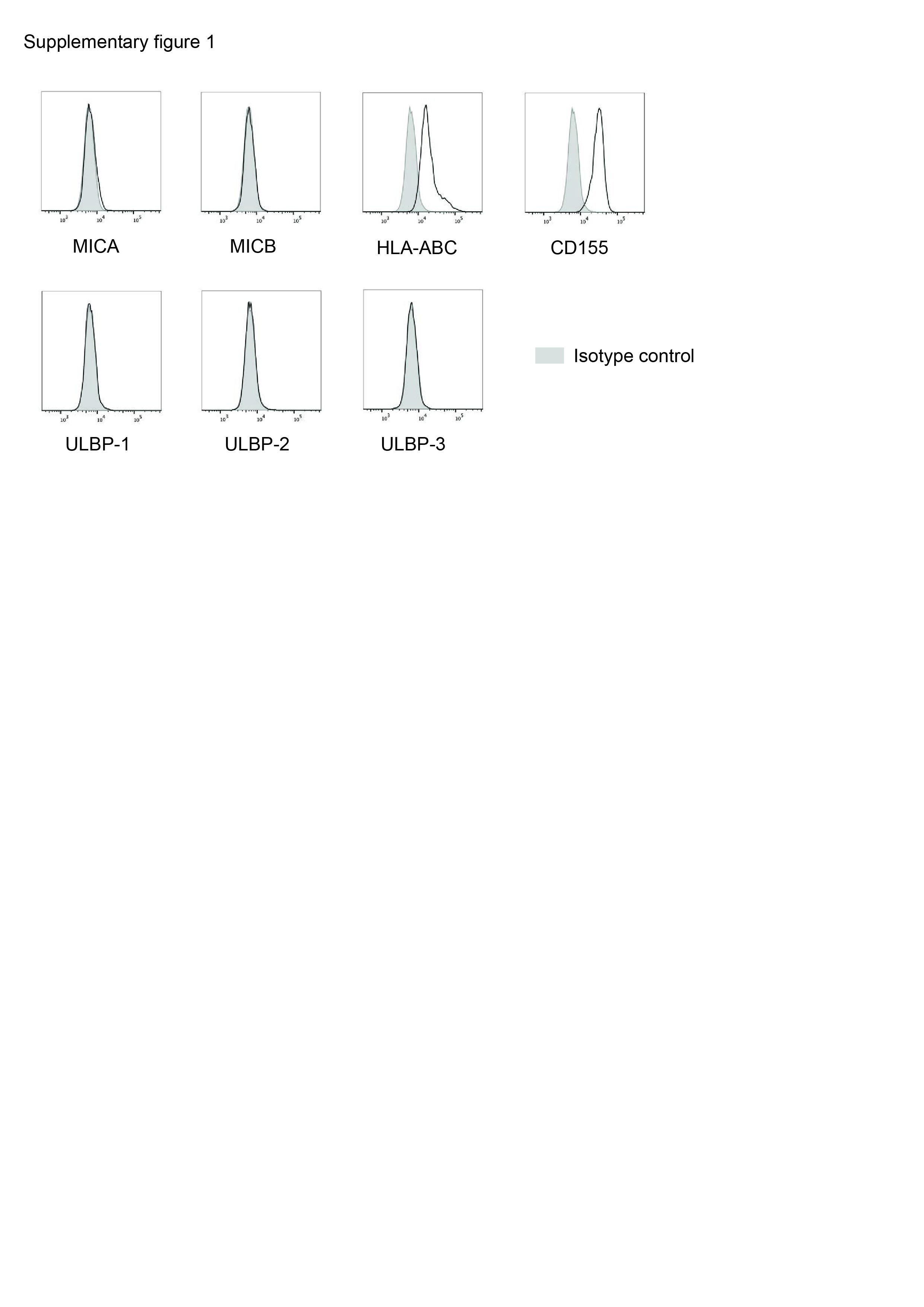

Supplement: Supplementary Figure 1 — Expression of NKG2D ligands on primary human hepatocytes. Representative histogram of flow cytometry stainings of NKG2D-ligands as well as HLA-ABC and CD155, ligand of DNAM-1, on primary hepatocytes. One representative staining out of three. [file Image_1.JPEG]
